# Data‐Driven Subtypes of Parkinson Disease Based on Dopamine Responsiveness

**DOI:** 10.1111/cns.70408

**Published:** 2025-04-24

**Authors:** Jian‐Yong Wang, Guo‐Ling Zeng, Rong‐Ting Tang, Hai‐Tao Luo, Yu‐Lian Song, Yang‐Yang Zhou, Jie‐Fan Huang, Shi‐Guo Zhu, Dao‐Lu Zhang, Dan‐Ni Liu, Rong‐Pei Liu, Shi‐Shi Huang, Cheng‐Xiang Yuan, Jian‐Hong Zhu, Xiong Zhang

**Affiliations:** ^1^ Department of Neurology and Institute of Geriatric Neurology, The Second Affiliated Hospital and Yuying Children's Hospital Wenzhou Medical University Wenzhou Zhejiang China; ^2^ Institute of Nutrition and Diseases and Center for Research, School of Public Health Wenzhou Medical University Wenzhou Zhejiang China; ^3^ Department of Nuclear Medicine, The Second Affiliated Hospital and Yuying Children's Hospital Wenzhou Medical University Wenzhou Zhejiang China

**Keywords:** dopamine responsiveness, heterogeneity, motor signs, Parkinson disease, subtypes

## Abstract

**Aims:**

Parkinson disease (PD) is highly heterogeneous in response to antiparkinsonian drugs. We herein aimed to identify PD subtypes based on dopamine responsiveness in three key motor signs (resting tremor, rigidity, and bradykinesia).

**Methods:**

The acute levodopa challenge test was performed. Improvement rates in resting tremor, rigidity, and bradykinesia were calculated. A total of 228 PD patients were included for further analysis. Subtypes were determined by k‐means clustering based on the improvement rates.

**Results:**

Four subtypes were identified: rt‐r‐b, moderate improvement in resting tremor, rigidity, and bradykinesia; RT‐r‐b, marked improvement in resting tremor but moderate improvement in rigidity and bradykinesia; RT‐R‐B, marked improvement in resting tremor, rigidity, and bradykinesia; rt‐R‐B, moderate improvement in resting tremor but marked improvement in rigidity and bradykinesia. These subtypes also differed in other motor and nonmotor symptoms.

**Conclusion:**

Our study reveals four distinct subtypes in PD patients based on dopamine responsiveness. Our findings provide a novel insight into understanding PD heterogeneity and facilitate precision treatment.

## Graphical abstract

Four subtypes are identified in Parkinson disease patients based on dopamine responsiveness.

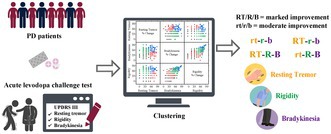

## 1 Introduction

Parkinson disease (PD) is a common neurodegenerative disease with resting tremor, rigidity, and bradykinesia as its key motor signs [1]. Besides the loss of dopaminergic neurons and the accumulation of Lewy bodies in the substantia nigra, abnormalities in other neurotransmitters such as acetylcholine, norepinephrine, and serotonin are also involved in PD pathology [2, 3, 4]. Dopamine replacement therapy compensates for the dopamine deficiency in the nigrostriatal system and remains the most effective pharmacological treatment for PD [5].

PD patients are highly heterogeneous in clinical manifestations and have been classified with different standards. Tremor dominant (TD) and postural instability and gait disorder (PIGD) are two classical subtypes based on motor symptoms [6]; juvenile‐onset, early‐onset, and late‐onset are subtypes classified based on age of onset [7]; and subtypes termed as “mainly motor/slow progression,” “diffuse/malignant,” and “intermediate” are defined on the basis of motor and nonmotor symptoms and disease progression rate [8]. Nevertheless, drug treatment response has not been applied in PD subtype classification, although its heterogeneity has been widely noted in the patients.

The acute levodopa challenge test is an easy and generally safe procedure. This test allows clinicians to observe the symptom improvement in a short time and has been frequently used in situations such as preoperative screening for deep brain stimulation, differential diagnosis from other Parkinsonian syndromes, and re‐evaluation of motor response [9]. Response to dopamine treatment differs significantly in PD patients, which brings a significant challenge in clinic for optimized pharmacotherapy. In this study, we recruited a cohort of PD patients for the acute levodopa challenge test and aimed to demonstrate whether the improvement rates of the three key motor signs can be used to define PD subtypes.

## 2 Materials and Methods

### 2.1 Patients

**FIGURE 1:**
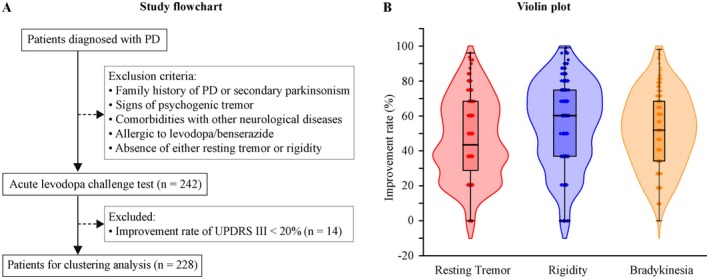
Study design and improvement rates in motor signs after the acute levodopa challenge test. (A) Flowchart of the study. (B) Violin plots showing the skewed distribution of improvement rates in resting tremor, rigidity, and bradykinesia. PD, Parkinson disease; UPDRS III, Unified Parkinson's Disease Rating Scale III.

PD patients were recruited from the Department of Neurology, the Second Affiliated Hospital, and Yuying Children's Hospital from August 2020 to June 2024. Patients were diagnosed according to the Movement Disorder Society clinical diagnostic criteria for PD established in 2015 [1]. The exclusion criteria for the acute levodopa challenge test were as follows: (1) family history of PD or secondary parkinsonism; (2) signs of psychogenic tremor; (3) comorbidities with other neurological diseases that may affect PD motor and nonmotor symptoms; (4) allergic to levodopa/benserazide; and (5) absence of either resting tremor or rigidity (Figure 1A). All participants signed written informed consents. This study was approved by the ethics committee of the Second Affiliated Hospital and Yuying Children's Hospital, Wenzhou Medical University (approval number LCKY2020‐278).

### 2.2 Clinical Evaluations

Age, gender, age at onset, and body mass index (BMI) were recorded. Clinical information was assessed by well‐trained investigators during “OFF” state (i.e., after a minimum 12‐h period without PD medication) [10], including Hoehn and Yahr stage, L‐dopa equivalent daily dosage (LEDD), Movement Disorder Society–Unified Parkinson's Disease Rating Scale (MDS‐UPDRS), Unified Parkinson's Disease Rating Scale (UPDRS) III, Mini‐mental State Examination (MMSE), Montreal Cognitive Assessment (MoCA), Non‐Motor Symptoms Scale (NMSS), Parkinson's Disease Questionnaire‐39 (PDQ‐39), Scales for Outcomes in Parkinson's Disease‐Automatic Symptoms (SCOPA‐AUT), REM Sleep Behavior Disorder Screening Questionnaire (RBD‐SQ), Hamilton Anxiety Scale (HAMA), and Hamilton Depression Scale (HAMD).

### 2.3 Acute Levodopa Challenge Test

The acute levodopa challenge test was performed during “OFF” state [11]. The challenge dose of levodopa/benserazide was 50% higher than the morning equivalent dose for the regularly medicated patients and was 200/50 mg for drug‐naive and irregularly medicated patients [12]. In brief, following evaluation of UPDRS‐III, the patient received 10 mg of domperidone and then the challenge dose of levodopa/benserazide. UPDRS‐III was reevaluated every 30 min for a period of up to 4 h. For each patient, the “ON” state was defined as the time point when his/her UPDRS‐III score was the lowest. The subscores including resting tremor (Item 20), rigidity (Item 22), and bradykinesia (Items 18, 19, 23, 24, 25, 26 and 31) in “OFF” and “ON” states were calculated, respectively [13]. The improvement rates in UPDRS‐III, resting tremor, rigidity, and bradykinesia were calculated using the formula: % change = 100 × [1–10^0.5 × (ON score–OFF score)^] [14]. Patients with an improvement rate of less than 20% in UPDRS III were excluded (Figure 1A). This rate is considered a negative result for the acute levodopa challenge test, suggesting that this patient may have atypical PD [12].

### 2.4 Statistical Analysis

Data were analyzed using Rstudio 2023.09.1 (https://posit.co/downloads/). Subtypes were determined by k‐means clustering based on the improvement rates in resting tremor, rigidity, and bradykinesia. The optimal number of clusters was determined by the Elbow method. Normality was assessed by the Kolmogorov–Smirnov test. Differences in age, duration, and age at onset were analyzed by the Kruskal–Wallis H test with the Benjamini–Hochberg test for correction of multiple comparisons. Differences in gender were evaluated by the Chi‐square test with Bonferroni correction for multiple comparisons. Other clinical characteristics were analyzed by a generalized linear model with disease duration, age, and gender as covariates and with Bonferroni correction for multiple comparisons. A two‐tailed *p* < 0.05 was considered statistically significant.

## 3 Results

### 3.1 Inclusion and Demographic and Clinical Characteristics of PD Patients

A total of 242 PD patients were initially included in the acute levodopa challenge test following the exclusion criteria. After the test, 14 patients with UPDRS III improvement rates of less than 20% were excluded from clustering analysis (Figure 1A). The remaining 228 PD patients comprised 121 females and 107 males. The age of the patients was 67.0 [interquartile range (IR) 61.0–73.0], the age at onset was 63.0 (IR 57.0–68.2), the MDS‐UPDRS score was 68.0 (IR 47.0–94.0), and the UPDRS III score was 39.0 (IR 25.8–54.0; Table 1). A total of 122 patients took medication regularly. Dopamimetic drugs varied among the patients and differed by time in some patients. LEDD (mg) for these patients was 375.0 (IR 249.8–600.0). Thirty‐nine patients took medication irregularly, and 67 patients had not taken any antiparkinsonian medication. Data for the duration of dopamimetic therapy were not consistently recorded and therefore were not analyzed. Following the acute levodopa challenge test, the improvement rate (%) was 50.5 ± 18.3 in UPDRS III, 43.4 (IR 32.8–68.4) in resting tremor, 60.2 (IR 36.9–74.9) in rigidity, and 51.9 (IR 34.2–68.4) in bradykinesia (Table 1; Figure 1). Among them, 25 patients developed levodopa‐induced dyskinesia, and 57 patients experienced wearing off. This relatively small size likely attributes to the overall short disease duration of the enrolled patients (median 3.0, IR 2.0–6.0).

**TABLE 1:** Demographic and clinical characteristics of PD patients.

Characteristics	Patients (*n* = 228)
Age (years), median (IR)	67.0 (61.0–73.0)
Gender, F/M	121/107
Duration (years), median (IR)	3.0 (2.0–6.0)
Age at onset (years), median (IR)	63.0 (57.0–68.2)
BMI, median (IR)	23.6 (21.4–25.4)
Hoehn and Yahr stage, median (IR)	2.0 (1.5–3.0)
LEDD^a^, mg (IR)	375.0 (249.8–600.0)
MDS‐UPDRS, median (IR)	68.0 (47.0–94.0)
MDS‐UPDRS III, median (IR)	44.0 (29.0–58.8)
UPDRS III, median (IR)	39.0 (25.8–54.0)
UPDRS III improvement rate (%), mean ± SD	50.5 ± 18.3
Resting tremor, median (IR)	4.0 (2.0–7.0)
Resting tremor improvement rate (%), median (IR)	43.4 (32.8–68.4)
Rigidity, median (IR)	9.0 (5.0–12.0)
Rigidity improvement rate (%), median (IR)	60.2 (36.9–74.9)
Bradykinesia, median (IR)	20.0 (12.0–27.0)
Bradykinesia improvement rate (%), median (IR)	51.9 (34.2–68.4)
MMSE, median (IR)	26.0 (22.0–29.0)
MoCA, median (IR)	20.0 (15.0–25.0)
NMSS, median (IR)	34.0 (20.5–57.5)
PDQ‐39, median (IR)	28.0 (17.0–43.8)
SCOPA‐AUT, median (IR)	9.0 (6.0–14.0)
RBD‐SQ, median (IR)	2.5 (1.0–5.0)
HAMA, median (IR)	9.5 (6.0–15.0)
HAMD, median (IR)	7.5 (4.0–13.0)

Abbreviations: BMI, body mass index; F, female; HAMA, Hamilton anxiety scale; HAMD, Hamilton depression scale; IR, interquartile range; LEDD, L‐dopa equivalent daily dosage; M, male; MDS‐UPDRS, Movement Disorder Society‐Unified Parkinson's Disease Rating Scale; MMSE, Mini‐mental State Examination; MoCA, Montreal Cognitive Assessment; NMSS, Non‐Motor Symptoms Scale; PD, Parkinson disease; PDQ‐39, Parkinson's Disease Questionnaire‐39; RBD‐SQ, REM Sleep Behavior Disorder Screening Questionnaire; SCOPA‐AUT, Scales for Outcomes in Parkinson's Disease‐Automatic Symptoms; SD, standard deviation; UPDRS, Unified Parkinson's Disease Rating Scale.

^a^ *n* = 122.

### 3.2 Cluster Analysis and Subtypes

**FIGURE 2:**
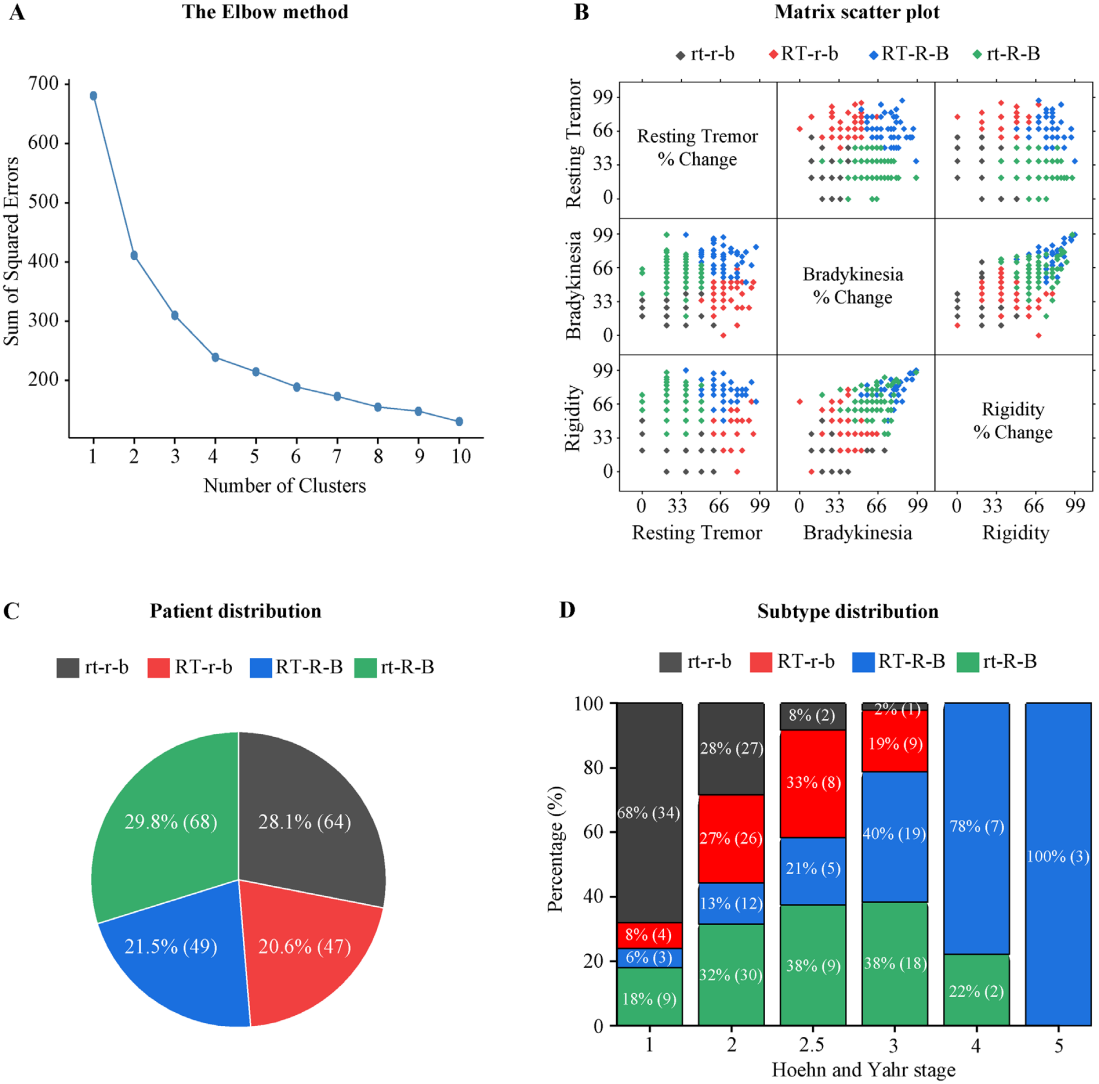
Cluster analysis of improvement rates in motor signs after the acute levodopa challenge test. (A) Optimal number of clusters determined by the Elbow method. (B) Matrix scatter plot showing distributions of improvement rates in resting tremor, rigidity, and bradykinesia in four clusters. (C) Patient distribution in the four clustered subtypes. (D) Subtype distribution in different Hoehn and Yahr stages. Numbers within (C) and (D) are proportions and numbers of patients. rt‐r‐b, patients with moderate improvement in resting tremor, rigidity, and bradykinesia; RT‐r‐b, patients with marked improvement in resting tremor but moderate improvement in rigidity and bradykinesia; RT‐R‐B, patients with marked improvements in resting tremor, rigidity, and bradykinesia; rt‐R‐B, patients with moderate improvement in resting tremor but marked improvement in rigidity and bradykinesia.

We then performed a cluster analysis on the improvement rates in different motor signs. The analysis determined four as the optimal number of clusters (Figure 2A). The improvement rates in resting tremor, rigidity, and bradykinesia were scattered in the four clusters as displayed by the matrices (Figure 2B). We defined the improvement rate between 20% and 50% as “moderate” and the improvement rate greater than 50% as “marked.” PD patients in Cluster 1 were characterized by moderate improvement in resting tremor, rigidity, and bradykinesia, termed as subtype rt‐r‐b; patients in Cluster 2 were characterized by marked improvement in resting tremor but moderate improvement in rigidity and bradykinesia, termed as subtype RT‐r‐b; patients in Cluster 3 were characterized by marked improvement in resting tremor, rigidity, and bradykinesia, termed as subtype RT‐R‐B; and patients in Cluster 4 were characterized by moderate improvement in resting tremor but marked improvement in rigidity and bradykinesia, termed as subtype rt‐R‐B (Table 2).

**TABLE 2:** Effect of levodopa on improvement rates of motor signs in four subtypes.

Cluster	Cluster 1	Cluster 2	Cluster 3	Cluster 4
Subtypes^a^	rt‐r‐b	RT‐r‐b	RT‐R‐B	rt‐R‐B
Patients, *n*	64	47	49	68
Resting tremor, mean improvement rate	31.6%	69.9%	66.7%	30.3%
Rigidity, mean improvement rate	29.1%	48.0%	78.4%	67.6%
Bradykinesia, mean improvement rate	32.4%	40.8%	74.4%	61.0%

Abbreviations: rb‐r‐b, patients with moderate improvement in resting tremor, rigidity, and bradykinesia; RT‐r‐b, patients with marked improvement in resting tremor but moderate improvement in rigidity and bradykinesia; RT‐R‐B, patients with marked improvement in resting tremor, rigidity, and bradykinesia; rt‐R‐B, patients with moderate improvement in resting tremor but marked improvement in rigidity and bradykinesia.

^a^ Capital and lowercase letters indicate marked (> 50%) and moderate (20%–50%) improvement rates, respectively.

Patients were distributed in the subtypes, with 28.1% in rt‐r‐b, 20.6% in RT‐r‐b, 21.5% in RT‐R‐B, and 29.8% in rt‐R‐B (Figure 2C). A further analysis of subtype distribution in different Hoehn and Yahr stages revealed that the rt‐r‐b proportion gradually decreased from Stages 1–5, while the RT‐R‐B proportion gradually increased. The RT‐r‐b and rt‐R‐B proportions were both higher in Stages 2 and 3 than in other stages (Figure 2D).

### 3.3 Comparison of the Subtypes in Clinical Characteristics

The four subtypes were comparable in age, gender, and age at onset (*p* > 0.05), but differed in disease duration (*p* < 0.001). The rt‐r‐b subtype had the shortest disease duration (median 2.0, IR 0.6–3.0), while the RT‐R‐B had the longest (median 6.0, IR 4.0–10.0). The RT‐r‐b and rt‐R‐B subtypes were comparable in disease duration. Differences of the subtypes in other clinical characteristics were then analyzed using disease duration, age, and gender as covariates (Table 3). Results showed that the four subtypes were comparable in BMI, but significantly differed in Hoehn and Yahr stage and PDQ‐39 (*p* < 0.001). The rt‐r‐b subtype had the earliest Hoehn and Yahr stage (median 1.5, IR 1.0–2.0), while the RT‐R‐B had the latest (median 3.0, IR 2.0–3.0). The rt‐r‐b contained the lowest PDQ‐39 score (median 17.0, IR 11.0–26.0).

**TABLE 3:** Comparison of clinical characteristics among the subtypes.

	rt‐r‐b	RT‐r‐b	RT‐R‐B	rt‐R‐B	*p*	*p*_1 vs. 2_ ^a^	*p*_1 vs. 3_ ^a^	*p*_1 vs. 4_ ^a^	*p*_2 vs. 3_ ^a^	*p*_2 vs. 4_ ^a^	*p*_3 vs. 4_ ^a^
Patients, *n*	64	47	49	68							
Characteristics											
Age (years), median (IR)	66.0 (59.0–70.2)	71.0 (64.0–74.0)	68.0 (60.0–72.0)	66.5 (61.0–73.0)	0.131^b^	0.107^c^	0.457^c^	0.505^c^	0.368^c^	0.356^c^	0.883^c^
Gender, F/M	33/31	26/21	28/21	34/34	0.864^d^	1.000^e^	1.000^e^	1.000^e^	1.000^e^	1.000^e^	1.000^e^
Duration (years), median (IR)	2.0 (0.6–3.0)	4.0 (2.0–7.0)	6.0 (4.0–10.0)	4.0 (2.0–5.0)	< 0.001^b^	< 0.001^c^	< 0.001^c^	< 0.001^c^	0.003^c^	0.437^c^	< 0.001^c^
Age at onset (years), median (IR)	63.5 (57.8–69.0)	64.0 (60.5–71.5)	62.0 (49.0–67.0)	61.0 (56.0–69.2)	0.106^b^	0.397^c^	0.232^c^	0.425^c^	0.179^c^	0.232^c^	0.397^c^
BMI, median (IR)	23.6 (21.3–25.3)	24.2 (22.1–25.9)	23.4 (20.8–24.9)	23.8 (21.5–25.7)	0.933^f^	1.000^e^	1.000^e^	1.000^e^	1.000^e^	1.000^e^	1.000^e^
Hoehn and Yahr stage, median (IR)	1.5 (1.0–2.0)	2.0 (2.0–2.5)	3.0 (2.0–3.0)	2.0 (2.0–3.0)	< 0.001^f^	< 0.001^e^	< 0.001^e^	< 0.001^e^	0.011^e^	1.000^e^	0.081^e^
PDQ‐39, median (IR)	17.0 (11.0–26.0)	36.0 (20.0–50.0)	43.0 (27.5–55.0)	29.0 (17.5–42.2)	< 0.001^f^	< 0.001^e^	0.001^e^	0.001^e^	1.000^e^	1.000^e^	1.000^e^
Motor symptoms											
MDS‐UPDRS, median (IR)	41.0 (27.3–56.8)	70.0 (52.0–103.0)	102.0 (84.0–123.0)	76.0 (61.3–90.3)	< 0.001^f^	< 0.001^e^	< 0.001^e^	< 0.001^e^	0.009^e^	1.000^e^	0.034^e^
MDS‐UPDRS III, median (IR)	23.5 (16.0–33.8)	44.0 (30.0–58.0)	63.0 (48.0–74.0)	52.0 (38.0–57.8)	< 0.001^f^	< 0.001^e^	< 0.001^e^	< 0.001^e^	0.003^e^	0.424^e^	0.274^e^
UPDRS III OFF, median (IR)	19.0 (13.0–27.0)	39.0 (28.0–51.5)	59.0 (53.0–65.0)	42.0 (34.0–49.0)	< 0.001^f^	< 0.001^e^	< 0.001^e^	< 0.001^e^	< 0.001^e^	0.694^e^	< 0.001^e^
UPDRS III ON, median (IR)	10.5 (6.0–17.0)	23.0 (13.0–33.5)	28.0 (18.0–35.0)	24.0 (13.8–32.2)	< 0.001^f^	< 0.001^e^	< 0.001^e^	< 0.001^e^	1.000^e^	1.000^e^	1.000^e^
UPDRS III improvement rate (%), mean ± SD	29.8 ± 8.3	48.8 ± 8.8	73.7 ± 9.6	54.5 ± 11.2	< 0.001^f^	< 0.001^e^	< 0.001^e^	< 0.001^e^	< 0.001^e^	0.042^e^	< 0.001^e^
Resting tremor OFF, median (IR)	3.0 (2.0–4.0)	8.0 (6.0–9.5)	6.0 (4.0–10.0)	2.0 (2.0–4.0)	< 0.001^f^	< 0.001^e^	< 0.001^e^	0.094^e^	0.322^e^	< 0.001^e^	< 0.001^e^
Resting tremor ON, (IR)	1.0 (1.0–2.3)	2.0 (1.0–4.0)	1.0 (0–4.0)	1.0 (0–2.0)	0.074^f^	1.000^e^	1.000^e^	0.869^e^	1.000^e^	0.093^e^	1.000^e^
Resting tremor improvement rate (%), median (IR)	36.9 (20.6–36.9)	68.4 (60.2–77.5)	68.4 (60.2–74.9)	36.9 (20.6–36.9)	< 0.001^f^	< 0.001^e^	< 0.001^e^	1.000^e^	1.000^e^	< 0.001^e^	< 0.001^e^
Rigidity OFF, median (IR)	4.0 (2.0–6.0)	7.0 (5.0–11.5)	12.0 (10.0–16.0)	11.0 (9.0–13.0)	< 0.001^f^	< 0.001^e^	< 0.001^e^	< 0.001^e^	< 0.001^e^	0.002^e^	0.446^e^
Rigidity ON, median (IR)	2.0 (1.0–4.0)	4.0 (3.0–7.5)	6.0 (3.0–7.0)	5.0 (2.0–8.0)	< 0.001^f^	0.004^e^	0.005^e^	< 0.001^e^	1.000^e^	1.000^e^	1.000^e^
Rigidity improvement rate (%), median (IR)	36.9 (20.6–36.9)	49.9 (36.9–60.2)	80.0 (74.9–84.2)	68.4 (60.2–76.2)	< 0.001^f^	< 0.001^e^	< 0.001^e^	< 0.001^e^	< 0.001^e^	< 0.001^e^	0.333^e^
Bradykinesia OFF, median (IR)	9.0 (6.0–16.0)	19.0 (11.0–23.0)	28.0 (25.0–33.0)	23.0 (17.0–26.2)	< 0.001^f^	< 0.001^e^	< 0.001^e^	< 0.001^e^	< 0.001^e^	0.001^e^	0.170^e^
Bradykinesia ON, median (IR)	5.0 (3.0–8.0)	13.0 (6.0–18.0)	15.0 (10.0–19.0)	13.0 (7.8–18.0)	< 0.001^f^	< 0.001^e^	< 0.001^e^	< 0.001^e^	1.000^e^	1.000^e^	1.000^e^
Bradykinesia improvement rate (%), median (IR)	34.2 (18.9–40.7)	46.6 (34.2–51.9)	76.9 (64.9–81.3)	61.0 (51.9–71.5)	< 0.001^f^	0.004^e^	< 0.001^e^	< 0.001^e^	< 0.001^e^	< 0.001^e^	0.009^e^
Nonmotor symptoms											
MMSE, median (IR)	28.0 (24.0–29.0)	24.0 (20.0–28.5)	24.0 (19.0–28.0)	25.0 (23.0–28.0)	0.608^f^	1.000^e^	1.000^e^	1.000^e^	1.000^e^	1.000^e^	1.000^e^
MoCA, median (IR)	23.0 (17.5–26.0)	19.0 (14.0–24.0)	19.0 (15.0–24.0)	21.0 (16.0–24.0)	0.817^f^	1.000^e^	1.000^e^	1.000^e^	1.000^e^	1.000^e^	1.000^e^
NMSS, median (IR)	24.0 (13.5–38.5)	42.0 (25.0–65.0)	57.0 (27.0–72.0)	34.5 (21.8–50.0)	0.001^f^	0.018^e^	0.002^e^	0.153^e^	1.000^e^	1.000^e^	0.308^e^
SCOPA‐AUT, median (IR)	6.0 (4.0–10.0)	8.5 (5.6–12.0)	12.0 (9.0–17.8)	10.0 (6.0–14.0)	0.010^f^	1.000^e^	0.021^e^	0.125^e^	0.122^e^	1.000^e^	1.000^e^
RBD‐SQ, median (IR)	2.0 (0–4.0)	3.0 (0–5.0)	4.0 (2.0–5.0)	2.0 (1.0–5.0)	0.586^f^	1.000^e^	1.000^e^	1.000^e^	1.000^e^	1.000^e^	1.000^e^
HAMA, median (IR)	7.0 (5.0–12.0)	11.0 (8.5–18.5)	12.0 (7.0–16.0)	9.0 (5.0–13.5)	0.013^f^	0.010^e^	0.536^e^	1.000^e^	1.000^e^	0.193^e^	1.000^e^
HAMD, median (IR)	6.0 (3.5–9.0)	10.0 (6.0–15.0)	10.0 (7.0–16.0)	7.0 (4.0–11.5)	0.004^f^	0.011^e^	0.119^e^	1.000^e^	1.000^e^	0.072^e^	0.433^e^

Abbreviations: BMI, Body mass index; F, Female; HAMA, Hamilton anxiety scale; HAMD, Hamilton depression scale; IR, Interquartile range; M, male; MDS‐UPDRS, Movement Disorder Society‐Unified Parkinson's Disease Rating Scale; MMSE, Mini‐mental State Examination; MoCA, Montreal Cognitive Assessment; NMSS, Non‐Motor Symptoms Scale; PDQ‐39, Parkinson's Disease Questionnaire‐39; RBD‐SQ, REM Sleep Behavior Disorder Screening Questionnaire; RT‐r‐b, patients with marked improvement in resting tremor but moderate improvement in rigidity and bradykinesia; RT‐R‐B, patients with marked improvement in resting tremor, rigidity, and bradykinesia; rt‐R‐B, patients with moderate improvement in resting tremor but marked improvement in rigidity and bradykinesia; SCOPA‐AUT, Scales for Outcomes in Parkinson's Disease‐Automatic Symptoms; SD, Standard deviation; UPDRS, Unified Parkinson's Disease Rating Scale.

^a^ Comparison between subtypes, where 1, 2, 3, and 4 indicate rt‐r‐b, RT‐r‐b, RT‐R‐B, and rt‐R‐B, respectively.

^b^ Analyzed by Kruskal–Wallis H test.

^c^ Corrected by Benjamini–Hochberg test.

^d^ Analyzed by Chi‐square test.

^e^ Corrected by Bonferroni correction.

^f^ Analyzed by a generalized linear model with disease duration, age, and gender as covariates.

Except in resting tremor ON (*p* = 0.074), the four subtypes significantly differed in all other motor symptom indicators (*p* < 0.001), probably because the cluster analysis was based on the improvement rates in motor signs. Among the subtypes, the rt‐r‐b had the lowest scores and the RT‐R‐B had the highest score in most of the motor symptom indicators. For nonmotor symptoms, the four subtypes differed in NMSS (*p* = 0.001), SCOPA‐AUT (*p* = 0.010), HAMA (*p* = 0.013), and HAMD (*p* = 0.004), but not in MMSE, MoCA, and RBD‐SQ. The rt‐r‐b carried lower scores than the other subtypes in these nonmotor symptom indicators (Table 3).

## 4 Discussion

Response to drug treatment differs significantly in PD patients. We herein identified four PD subtypes based on the response of three key motor signs to acute dopamine challenge, that is, rt‐r‐b (moderate improvement in resting tremor, rigidity, and bradykinesia), RT‐r‐b (marked improvement in resting tremor but moderate improvement in rigidity and bradykinesia), RT‐R‐B (marked improvement in resting tremor, rigidity, and bradykinesia), and rt‐R‐B (moderate improvement in resting tremor but marked improvement in rigidity and bradykinesia). These four subtypes also display distinguishing features in other clinical characteristics.

Previous PD subtypes primarily reflect age of onset, clinical features, neuroimaging, and molecular markers [15, 16, 17]. Levodopa is the most effective symptomatic treatment drug for PD. However, a small number of patients benefit little due to limited responsiveness to this drug [18, 19]. Identifying this subtype of PD patients is valuable in clinical practice. Dopamine responsiveness has been previously used to classify PD patients, but only resting tremor was assessed [14]. In this study, we comprehensively evaluated dopamine responsiveness of three key motor signs and employed a cluster analysis to differentiate PD patients. The achieved four subtypes display unique features: The RT‐R‐B subtype has the best dopamine responsiveness, with the most promising improvement of overall motor symptoms and some nonmotor symptoms; the RT‐r‐b and rt‐R‐B subtypes are in the middle, with RT‐r‐b showing better improvement in resting tremor and rt‐R‐B showing better improvement in rigidity and bradykinesia; and the rt‐r‐b subtype is on the other end, with the worst responsiveness to dopamine treatment. Nondopaminergic drugs such as benzhexol and amantadine are thus recommended as early as possible to treat these patients.

The four subtypes are distinct in disease duration, Hoehn and Yahr stage, PDQ‐39, MDS‐UPDRS, MDS‐UPDRS III, and UPDRS III OFF. In general, the rt‐r‐b subtype is in the earlier stage and carries relatively mild symptoms, while the RT‐R‐B subtype, which responds the best to dopamine treatment, is in a more advanced stage and bears more severe symptoms. Indeed, dopamine responsiveness may become more pronounced as the disease progresses. Meanwhile, there are additional merits implicated in this subtyping. First, the clustering of RT‐r‐b and rt‐R‐B is unique, which reveals that rigidity and bradykinesia respond similarly to dopamine treatment while resting tremor responds separately. In line with this finding, the disrupted nigrostriatal dopaminergic system has been reported to be more closely linked to bradykinesia and rigidity than resting tremor [20, 21]. Neurotransmitters such as serotonin and noradrenaline may additionally contribute to resting tremor and its dopamine response [4, 22, 23]. Second, the four subtypes also display distinct characteristics in nonmotor symptoms, autonomic symptoms, anxiety, and depression, although they are classified based on dopamine responsiveness. Patients of RT‐R‐B, RT‐r‐b, and rt‐R‐B may require increased attention to these symptoms. Third, the subtype distribution gradually changes in Hoehn and Yahr stages, suggesting that a patient's subtype may change as the disease progresses. Notably, changes in PD motor subtypes of tremor‐dominant, indeterminate, and postural instability gait difficulty have been previously demonstrated [24]. Changes in our subtypes warrant further investigation in longitudinal studies.

The prevalence of PD is higher in men than in women by a ratio of approximately 1.4 [25]. However, our study has more women enrolled, probably due to a higher willingness in women to participate and a higher prevalence of cerebrovascular disease in men [26]. The latter may lead to more men being excluded as a comorbidity. Meanwhile, several factors should be considered when interpreting our results. First, we herein only assessed patients' response to levodopa. It is possible that some patients respond poorly to levodopa but respond well to other medications [27]. Second, a small portion of PD patients lacks resting tremor or rigidity [1]. These patients were not included in our study, which otherwise may lead to more complicated clustering results. Therefore, the current classification is only applicable to PD patients carrying three key motor signs. Third, most of the patients included herein were in Hoehn and Yahr Stages 1–3, and only 12 patients were in Stages 4–5. Drug response in advanced stage is often more complicated. Therefore, further studies with more advanced PD patients are needed to validate this classification.

In summary, the present study reveals four distinct subtypes in PD patients based on dopamine responsiveness. Our findings provide a novel insight into understanding the heterogeneity of PD and may facilitate precision treatment for this disease.

## Author Contributions

J.Y.W. and X.Z. conceived the main idea; J.Y.W., G.L.Z., J.F.H., and S.G.Z., performed the challenge test; G.L.Z., J.F.H., D.N.L., R.P.L., S.S.H. and C.X.Y. collected clinical data; J.Y.W., G.L.Z., R.T.T., H.T.L., Y.L.S., Y.Y.Z., and D.L.Z. analyzed and interpreted data; J.Y.W. and G.L.Z. drafted the manuscript; J.H.Z. and X.Z. edited the manuscript. All authors have read and approved the final manuscript.

## Ethics Statement

This study was approved by the ethics committee of the Second Affiliated Hospital and Yuying Children's Hospital, Wenzhou Medical University.

## Consent

All participants signed written informed consent.

## Conflicts of Interest

The authors declare no conflicts of interest.

## Data Availability

The data that support the findings of this study are available from the corresponding author upon reasonable request.
